# Molecular engineering of the salicylate-inducible transcription factor Sal7AR for orthogonal and high gene expression in *Escherichia coli*

**DOI:** 10.1371/journal.pone.0194090

**Published:** 2018-04-11

**Authors:** Kentaro Miyazaki

**Affiliations:** 1 Bioproduction Research Institute, National Institute of Advanced Industrial Science and Technology (AIST), Tsukuba, Ibaraki, Japan; 2 Department of Computational Biology and Medical Sciences, Graduate School of Frontier Sciences, The University of Tokyo, Kashiwa, Chiba, Japan; Dong-A University, REPUBLIC OF KOREA

## Abstract

I have previously identified a metagenomic fragment (~4 kb) containing the salicylate (2-hydroxybenzoate)-responsive transcriptional regulator Sal7AR. Taking advantage of the inert nature of salicylate to common genetic switches used in *Escherichia coli*, here I developed a salicylate-inducible high expression system in *E*. *coli*. I first applied a deletion analysis to the metagenomic fragment to identify the core region (~1 kb) necessary for the salicylate-dependent expression. Sal7AR was subjected to an error-prone PCR, and a library was screened for an enhanced expression of a reporter green fluorescent protein (GFP) gene in the presence of 1 mM salicylate, where virtually no growth inhibition was observed. Three beneficial amino acid substitutions were identified (N282K, Q292R, and V295G), each of which improved the expression of GFP relative to the wildtype by several-fold. The three sites were then completely randomized by saturation mutagenesis either individually or combinatorially to identify three variants carrying a single point mutation, N282L, V295F, or V295S; no further improvements were observed by combining these mutations. Salicylate-dependent expression of these mutants was highly repressed in its absence and escalated in response to ~10 μM salicylate, and gradually increased up to 1 mM salicylate; the induction rate was approximately 15 times greater than that achieved with a lactose promoter. Orthogonality to the lactose-based expression system was also confirmed. This salicylate-based expression system should thus be advantageously used for high-level production of recombinant proteins in combination with common lactose-dependent induction systems.

## Introduction

*Escherichia coli* is the most widely used host microorganism for heterologous gene expression purposes, in which promoters play a crucial role and various promoters have been developed that respond to a specific compound as an inducer. To date, the most common inducible promoters are T7, T5, lactose, and tac (tryptophan/lacUV5), all of which rely on a LacI-negative transcription regulator and the expression is commonly induced by isopropyl β-D-1-thiogalactopyranoside (IPTG), a non-metabolizable analogue of lactose. However, despite the variety of promoters, it is not possible to independently regulate each promoter in the same cell.

Apart from the lactose-based induction system, there are a couple of sugar-based expression systems; P_BAD_-based expression is activated by L-arabinose and highly repressed in its absence. One drawback in the P_BAD_ system is that L-arabinose is readily metabolized in *E*. *coli*, and non-metabolized analogues are not available to date; for continuous induction, a special mutant strain, which lacks the ability to assimilate L-arabinose, is required as a host. To complement the drawback in the P_BAD_ system, the AraC transcriptional regulator was engineered such that it specifically recognizes chiral D-arabinose as an inducer, which is not metabolized in *E*. *coli* cells [1]. Similarly, Kelly et al. (2016) have succeeded in converting the specificity of the transcriptional regulator RhaS, which originally responds to the metabolizable sugar L-rhamnose, such that it responds to L-mannose, a non-metabolizable sugar in *E*. *coli* [2]. In this synthetic biology era, there is a need to independently regulate multiple genes (or gene clusters) in the same cell; thus, it is necessary to develop as many genetic switches as possible with induction specificities independent from one another. It is also preferable that the induction agents are metabolically stable in standard *E*. *coli* strains for continuous induction. For scalable production, the cost of the agents is also a matter for consideration.

Bacteria that degrade aromatic compounds are widespread in natural and polluted environments [3]. These degrading bacteria contain various types of degradation pathways [4], whose expressions are often activated by transcription factors with a cognate metabolite as a co-inducer. Although such degraders are widespread in the environment, *E*. *coli* usually lacks the ability to sense and degrade such aromatic compounds [5–8]. Previously, through a functional (promoter activity) screen of a metagenomic library, we have isolated several clones that contained transcription factors that were responsive to aromatic compounds [5, 7]. One of the clones, pSAL7A, was highly specific to salicylate (2-hydroxybenzoate), but the induction rate was poor [8]. I considered that the transcriptional regulator, Sal7AR included in pSAL7A, would be potentially useful to develop a novel genetic switch. The advantages of using salicylate as an induction agent was primarily because of its chemical orthogonality to common sugar-based induction systems and its stability; the compound was not metabolized in standard laboratory strains of *E*. *coli* [9], was chemically stable under normal growth conditions, and thus long-lasting induction can be anticipated. High permeability was also of great importance such that no additional gene(s) would be needed to transport the compound (such as *lacY* in lactose operon). Finally, the low cost of the compound was also advantageous. One factor that needs to be taken into consideration is the potential toxicity because salicylate is widely used as an antiseptic/preservative agent in industry; *E*. *coli* is susceptible to salicylate and thus growth is perturbed at > 10 mM salicylate [9–11]. Therefore, it is necessary that the transcription factor is capable of recognizing low concentrations of salicylate where no growth inhibition occurs. Some metabolic (or transcriptomic and proteomic) changes are induced at high concentrations (~10 mM) [9–11], but the induction condition for Sal7AR is well below that concentration (1 mM) and thus those effects would be negligible.

In this study, various modifications were made for the pSAL7A vector, first by trimming unnecessary regions from the metagenomic insert, followed by engineering Sal7AR via directed evolution. Error-prone PCR and subsequent saturation mutagenesis [12] identified variants with a ~20-fold enhanced induction rate relative to the original pSAL7A, which was ~15-fold that achieved by the lactose promoter. The GFP-expressing vector (ColE1 ori, ampicillin [Amp] resistant) was co-introduced into *E*. *coli* JM109 with a compatible pACYC-based vector (p15a ori, chloramphenicol [Cm] resistant) expressing *E*. *coli* alkaline phosphatase. Dose-dependent independent expression was observed for these two proteins, confirming the orthogonality of the salicylate-based induction from the IPTG-based induction. The salicylate-inducible expression system developed in this study could thus be advantageously used for synthetic biology.

## Materials and methods

### Reagents

T4 polynucleotide kinase, T4 DNA ligase, and DpnI were purchased from New England Biolabs (Piscataway, NJ). Oligonucleotide primers were purchased from Eurofin Genomics (Tokyo, Japan). GeneMorph Random Mutagenesis Kit was purchased from Stratagene (La Jolla, CA). KOD plus Neo DNA polymerase was purchased from Toyobo (Osaka, Japan). In-Fusion Cloning Kit and pSTV28 were purchased from Takara Bio (Shiga, Japan). Lennox LB powder (1% [w/v] tryptone, 0.5% [w/v] yeast extract, and 0.5% [w/v] NaCl) and agar powder were purchased from Merck (Darmstadt, Germany). Antibiotics (Amp and Cm) and *p*-nitrophenyl phosphate were purchased from Wako Pure Chemicals (Tokyo, Japan). Salicylic acid was purchased from Tokyo Kasei (Tokyo, Japan); 1 M stock solution was prepared in dimethyl sulfoxide. All other reagents were of the purest grade available.

### Bacterial strains and media

*E*. *coli* JM109 was used throughout this study as an expression host and was routinely grown in Lennox LB at 37°C. Competent *E*. *coli* JM109 cells (*recA1 endA1 gyrA96 thi-1 hsdR17 supE44 relA1* Δ*(lac-proAB*) /F’ [*tra36 proAB*^*+*^ *lacI*^*q*^ *lacZ*Δ*M15*]) were purchased from RBC Bioscience (Taipei, Taiwan). Amp was used at 100 μg/ml, Cm was at 34 μg/ml, and salicylate was at 1 mM if not otherwise stated. Agar was added to solidify the medium at 1.6% (w/v).

### Construction of the deletion mutants

**Fig 1:**
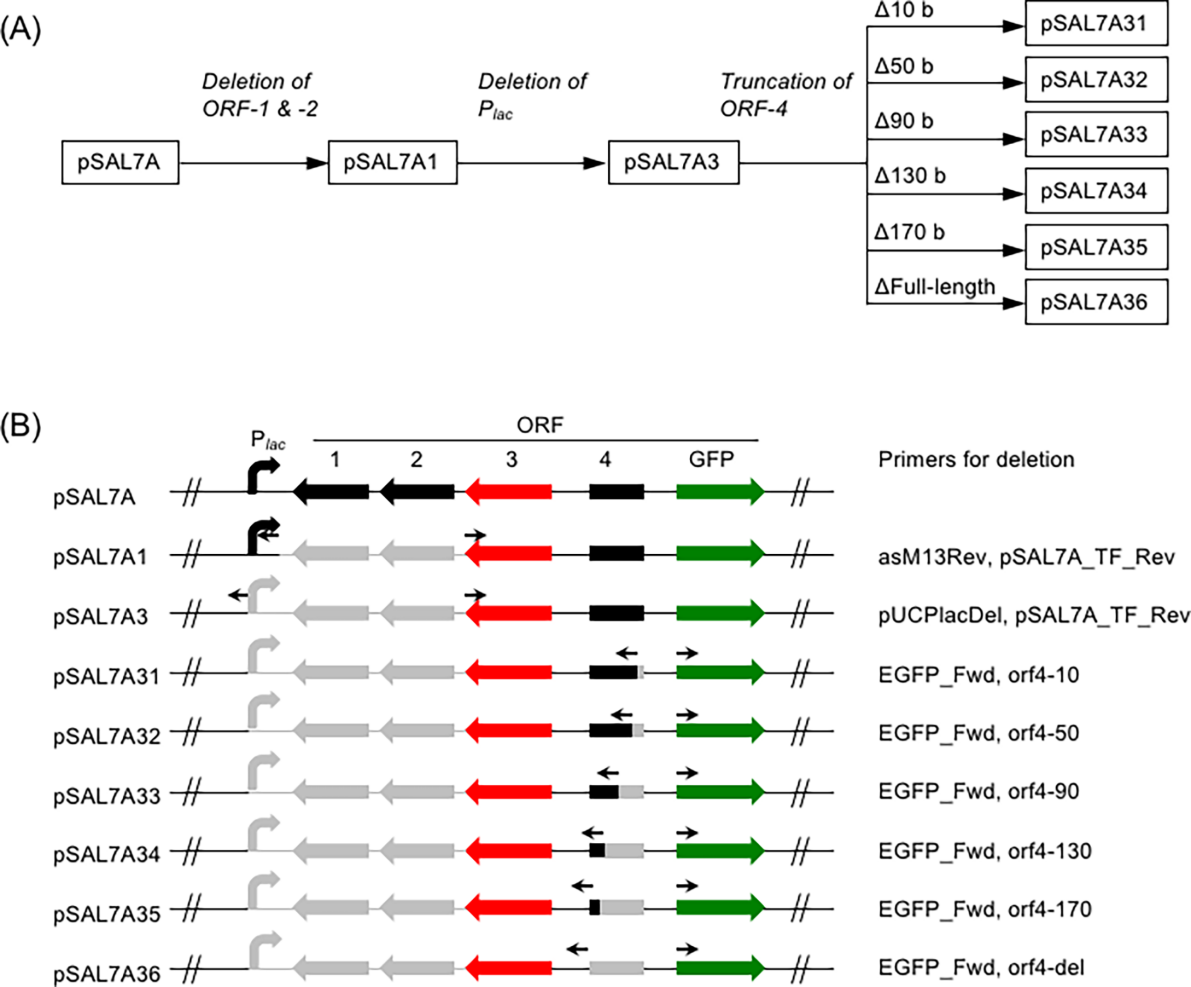
Schematic representation for the construction of the deletion mutants. (A) Flow chart. (B) Series of deletion mutants and primers used for inverse PCR. Parts drawn in gray indicate the deleted regions. Primers are illustrated with opposing arrows.

Inverse PCR was conducted to delete the target region from the template vectors (pSAL7A and its derivatives). The template and a set of primers are summarized in Fig 1 and the oligonucleotide primer sequences are listed in S1 Table. In all reactions, the PCR mixture contained 1× PCR buffer, 0.2 μM of each dNTP, 50 ng of template DNA, 0.25 mM of each primer, and 0.5 U of KOD-Plus-Neo DNA polymerase in a total volume of 25 μl. The mixture was heated at 98°C for 2 min, and subjected to 25 cycles of incubation at 98°C for 10 s, 57°C for 30 s, and 72°C for 2 min, and a final incubation at 68°C for 5 min. After the reaction, DpnI (5 U) was added to the products and incubated at 37°C overnight. The reaction products were separated by 0.9% (w/v) agarose gel electrophoresis, gel-purified, and self-ligated using T4 polynucleotide kinase and T4 DNA ligase by incubating at 25°C for 2 h. Competent *E*. *coli* JM109 cells were transformed with the mixture and grown on LB/Amp agar plates at 37°C. Several colonies were picked, and plasmids carrying the designed construct were identified by DNA sequencing.

### Error-prone PCR

Random mutagenesis was carried out on an error-prone PCR procedure using a set of primers, TF_5UTR and pUCPlacDelas (S1 Table), which enabled the amplification of the entire *sal7AR* gene. The reaction mixture contained 1× PCR buffer, 0.2 mM of each dNTP, 0.1 μM of each primer, 1 μg of pSAL7A35 (template) (Fig 1), and 2.5 U of Mutazyme II DNA polymerase in a total volume of 50 μl. The mixture was heated at 95°C for 2 min, followed by 25 cycles of incubation at 95°C for 10 s, 57°C for 30 s, and 72°C for 1 min, and a final incubation at 72°C for 10 min. After cooling, DpnI (5 U) was directly added to the products and incubated at 37°C overnight. The reaction products were separated by agarose gel electrophoresis and the insert fragment (~1 kb) was gel-purified and dissolved in 30 μl of water.

To clone back the fragment into the pSAL7A35 vector, we used the In-Fusion method. To this end, pSAL7A35 was inversely amplified using a set of primers, TF_5UTRas and pUCPlacDel, to prepare the linear vector lacking the *sal7AR* gene. The PCR mixture contained 1× PCR buffer, 0.2 mM of each dNTP, 50 ng of pSAL7A35 (template), 0.25 μM each of primers, and 0.5 U of KOD-Plus-Neo DNA polymerase in a total volume of 50 μl. The mixture was heated at 98°C for 2 min, and subjected to 25 cycles of incubation at 98°C for 10 s, 57°C for 30 s, and 68°C for 2.5 min, and a final incubation at 68°C for 5 min. After cooling, DpnI (5 U) was directly added to the products and incubated at 37°C overnight. The reaction products were separated by agarose gel electrophoresis and the vector fragment (~3.6 kb) was excised from the gel, purified and dissolved in 30 μl of water. The insert and vector fragments (2 μl each) were combined with the In-Fusion reagent to give a final volume of 10 μl, and then the tube was incubated at 50°C for 1 h. A portion of the sample was used to transform competent *E*. *coli* JM109 cells. Transformants were selected on LB/Amp agar plates containing 1 mM salicylate (LB/Amp/SAL).

### Site-saturation mutagenesis

Saturation mutagenesis [12] was carried out using a set of complementary primers as listed in S1 Table. The target amino acid position was coded by NNK; where N was A, G, C, or T; and K was G or T. A pair of primers was mixed in 1× PCR buffer, 0.2 mM of each dNTP, 50 ng of pSAL7A35 (template), 0.25 mM of each primer, and 0.5 U of KOD-Plus-Neo in a total volume of 25 μl. The mixture was heated at 98°C for 2 min followed by 30 cycles of incubation at 98°C for 10 s, 57°C for 30 s, and 68°C for 2.5 min, and a final incubation at 68°C for 5 min. After the reaction, DpnI (5 U) was then directly added to the products and incubated at 37°C overnight. The amplicon was gel-purified and self-ligated using T4 polynucleotide kinase and T4 DNA ligase. A portion of the sample was used to transform competent *E*. *coli* JM109 cells, and transformants were grown on LB/Amp/SAL agar plates.

### Library screening

Library screening was carried out based on fluorescence derived from GFP. Colonies appeared on LB/Amp/SAL plates were exposed to blue light (Invitrogen, Safe Imager 2.0), and bright colonies were visually identified. They were then grown in LB/Amp broth at 37°C overnight in separate square wells of a 2-ml 96 deep-well plate. The pre-culture (1 μl) was then transferred to 1 ml of fresh LB/Amp and LB/Amp/SAL broth and grown at 37°C overnight with vigorous shaking (1,200 rpm). Next, 170 μl of the culture was transferred to a 96-well plate (black bottom), and fluorescence was measured in a spectrofluorometer (Spectramax Gemini XS, Molecular Devices, Sunnyvale, CA, USA) at excitation and emission wavelengths of 488 nm and 530 nm, respectively.

### Alkaline phosphatase activity

The activity was measured in 100 mM Tris-HCl (pH 10), containing 1 mM MgCl_2_ and 1 mM *p*-nitrophenyl phosphate. The initial rate of activity was monitored for 5 min at room temperature in a Molecular Devices (Sunnyvale, CA, USA) spectrometer (Versa Max) at 410 nm.

## Results and discussion

### Deletion analysis

The pSAL7A vector contains a metagenomic insert fragment of approximately 4 kb in length from the promoter-trapping vector p18GFP [5], in which *orf3* codes for the salicylate-responsive transcription factor [8] (Fig 1). The transcription factor, designated Sal7AR, is highly specific to salicylate (2-hydroxybenzoate); virtually no cross-reactivity was observed for the following aromatic compounds: phenol, catechol, o-cresol, m-cresol, p-cresol, 3-methylcatechol, 4-methylcatechol, 4-chlorocatechol, benzoate, 3-hydroxybenzoate, 4-hydroxybenzoate, 2-chlorobenzoate, 3-chlorobenzoate, 4-chlorobenzoate, hydroquinone, chlorohydroquinone, and 4-chlororesorcinol [8]. Although ORF3 has been clarified to be responsible for the salicylate-dependent induction [8], it remains to be elucidated how flanking regions affect the induction activity. To identify the core regions necessary for the expression, I first employed a deletion analysis. The overall scheme used to construct the deletion mutants is illustrated in Fig 1.

### Deletion of *orf-1* and *orf-2*

**Fig 2:**
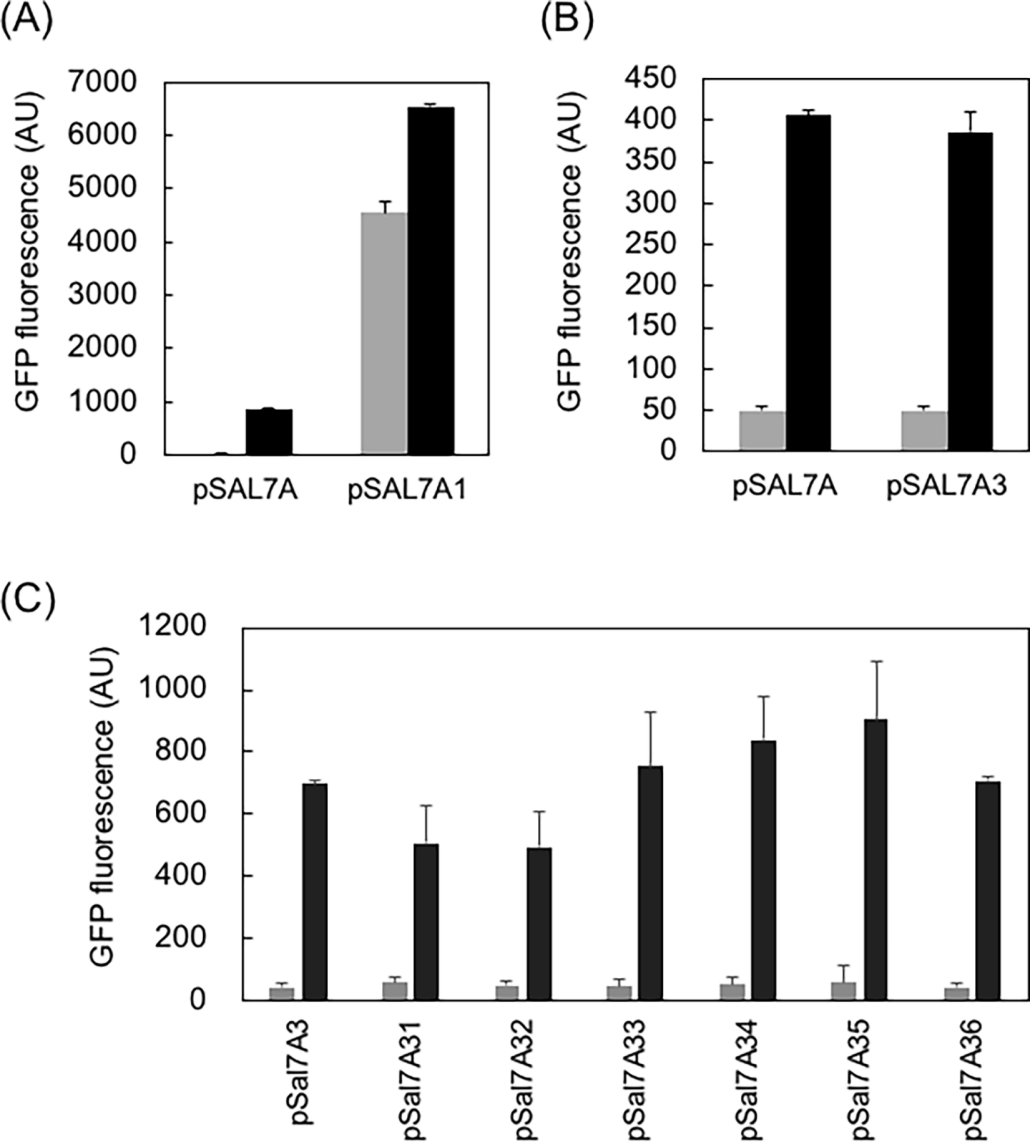
Fluorescence intensity of deletion mutants. (A) Metagenomically retrieved parental pSAL7A [8] and pSAL7A1 lacking ORFs -1 and -2. (B) pSAL7A [8] and pSAL7A3 lacking ORFs -1 and -2, and P_*lac*_. (C) pSAL7A3 and a series of ORF-4 deletion mutants (pSAL7A31–36). *E*. *coli* JM109 cells were grown in LB/Ampicillin at 37°C for 16 h. *N* = 4. Grey bars: 0 mM salicylate; Black bars: 1 mM salicylate.

First, I deleted two open reading frames, ORF-1 and ORF-2, based on our previous observation that these ORFs did not affect (positively nor negatively) the induction [8]. A set of primers, asM13Rev and pSAL7A_TF_Rev (primer sequences summarized in S1 Table, were used to inversely amplify pSAL7A. The linear fragment was then self-ligated to yield pSAL7A1. A large increase in the expression was observed, which was, however, independent of salicylate (Fig 2A).

### Deletion of the lactose promoter/operator

I considered that the salicylate-independent expression observed in pSAL7A1 was caused by leaky expression from the lac promoter (P_*lac*_). I thus deleted P_*lac*_ and its operator O_*lac*_ from pSAL7A1. A set of primers, pUCPlacDel and TF_Rev, was used to remove the lac promoter/operator region from pSAL7A1 to generate the recombinant vector pSAL7A3. The salicylate dependent expression was restored and the induction rate was equivalent to the parental pSAL7A vector (Fig 2B).

### Deletion of *orf-4*

I next deleted the 5’ upstream region of the reporter *gfp* gene. The *orf4* gene codes for truncated gene product (64 aa), which showed the highest similarity (88%) to naphthalene 1,2-dioxygenase from Burkholderiales bacterium. A series of truncation variants were constructed by systematic deletion using a set of primers consisting of GFP_Eco_Fwd with either orf4-10, orf4-50, orf4-90, orf4-130, orf4-170, orf4-del. Virtually no leaky expression was observed in the absence of salicylate (Fig 2C, grey bars), and the highest fluorescence was obtained for orf4-170 in the presence of 1 mM salicylate (Fig 2C, black bars); the resulting plasmid was named pSAL7A35, which was then used for further studies.

### Directed evolution of SAL7AR

#### Error-prone PCR

**Fig 3:**
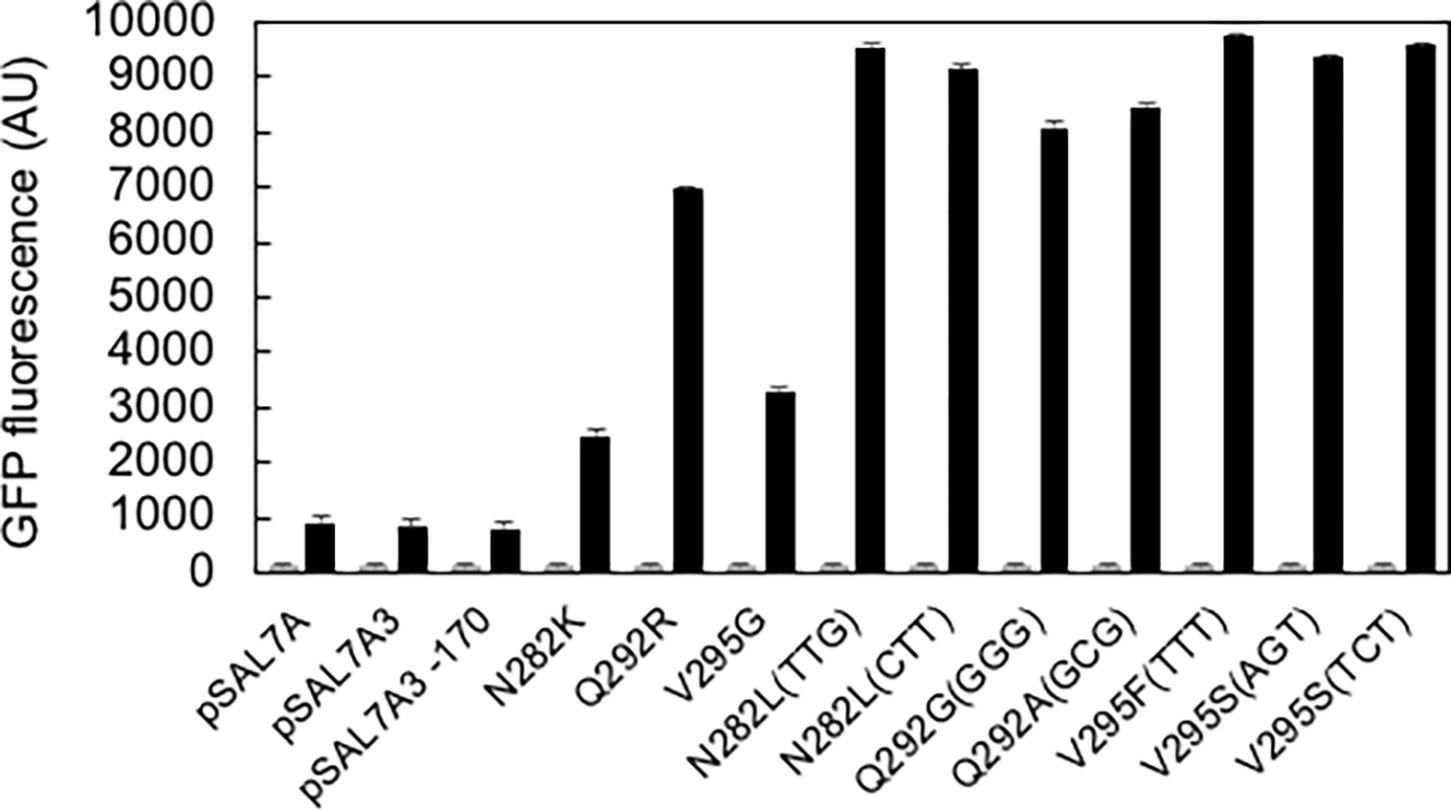
Fluorescence intensity of variants obtained from the error-prone PCR library. *E*. *coli* JM109 cells were grown in LB/Ampicillin at 37°C for 16 h. *N* = 4. Grey bars: 0 mM salicylate; Black bars: 1 mM salicylate.

Error-prone PCR was employed to randomize the *sal7a* gene (915 bases) to generate an average of 3–4 errors in the gene. The resultant library (~40,000 clones) constructed in *E*. *coli* JM109 was screened on LB/Amp agar plates containing 1 mM salicylate. A total of 24 clones were visually selected under blue light, and then grown in LB/Amp broth in the presence and absence of salicylate for quantitative analysis. DNA sequencing of the top 10 variants revealed the presence of at least one amino acid replacement in each mutant (Table 1 and Fig 3, grey bars, uninduced [0 mM salicylate]; black bars, induced [1 mM salicylate]). Many of the variants shared the N282K mutation (A1, B2, B3, C3, D3, and E1), and many of the mutations (Q292R in A3 and V295A in C2) localized at the C-terminal region.

**Table 1:** Mutations identified in the selected clones from the error-prone PCR library.

Clone	Amino acid substitutions (Nucleotide substitutions)	Fluorescence (AU)	
		None	1 mM salicylate
WT	None	118	799
A1	Q237H (A711T), N282K (C846A)	114	2625
A3	V83I (G247A), P262P (T789C), Q292R (A875G)	137	6634
B2	L122F (C364T), N282K (C846G)	118	2716
B3	K29K (G87A), E214E (A641G), N282K (C846A)	120	3010
C1	D2N (G4A)	125	4484
C2	V295A (T884C)	129	2676
C3	E195E (G585A), V226L (T677C), V232V (T696C), N282K (C846G)	116	2675
D3	A243G (C728G), N282K (C846A)	118	2435
E1	L268L (C802T), N282K (C846A)	119	2886
F3	E117D (A351T), D210D (C630T), I266T (T797C)	122	2098

Values are the fluorescence intensity in the absence and presence of salicylate in LB/Amp after the growth at 37°C for 16 h.

#### Saturation mutagenesis

Mutation sites localized at the C-terminal region were next targeted by saturation mutagenesis. The amino acid positions 292 and 295 were singly or doubly mutated. Fig 3 summarizes the results of the screen. As for N282, two variants were selected, both of which contained Leu at the site with different codons, TTG and CTT; the former showed slightly higher fluorescence. As for Q292, two variants were selected that contained Gly (GGG) and Ala (GCG) at the site; with Q292A showing slightly higher fluorescence. As for V295, four variants were selected, that contained Phe (TTT x 2) or Ser (AGT, TCT) at the site; these showed nearly the same fluorescence. I also created a double-mutant library for sites 282 and 292, and 282 and 295, but no enhanced variants were obtained. Compared to the original pSAL7A (and its deletion derivatives pSAL7A3 and pSAL7A35), a large increase (approximately 15-fold) in the expression level was achieved.

**Fig 4:**
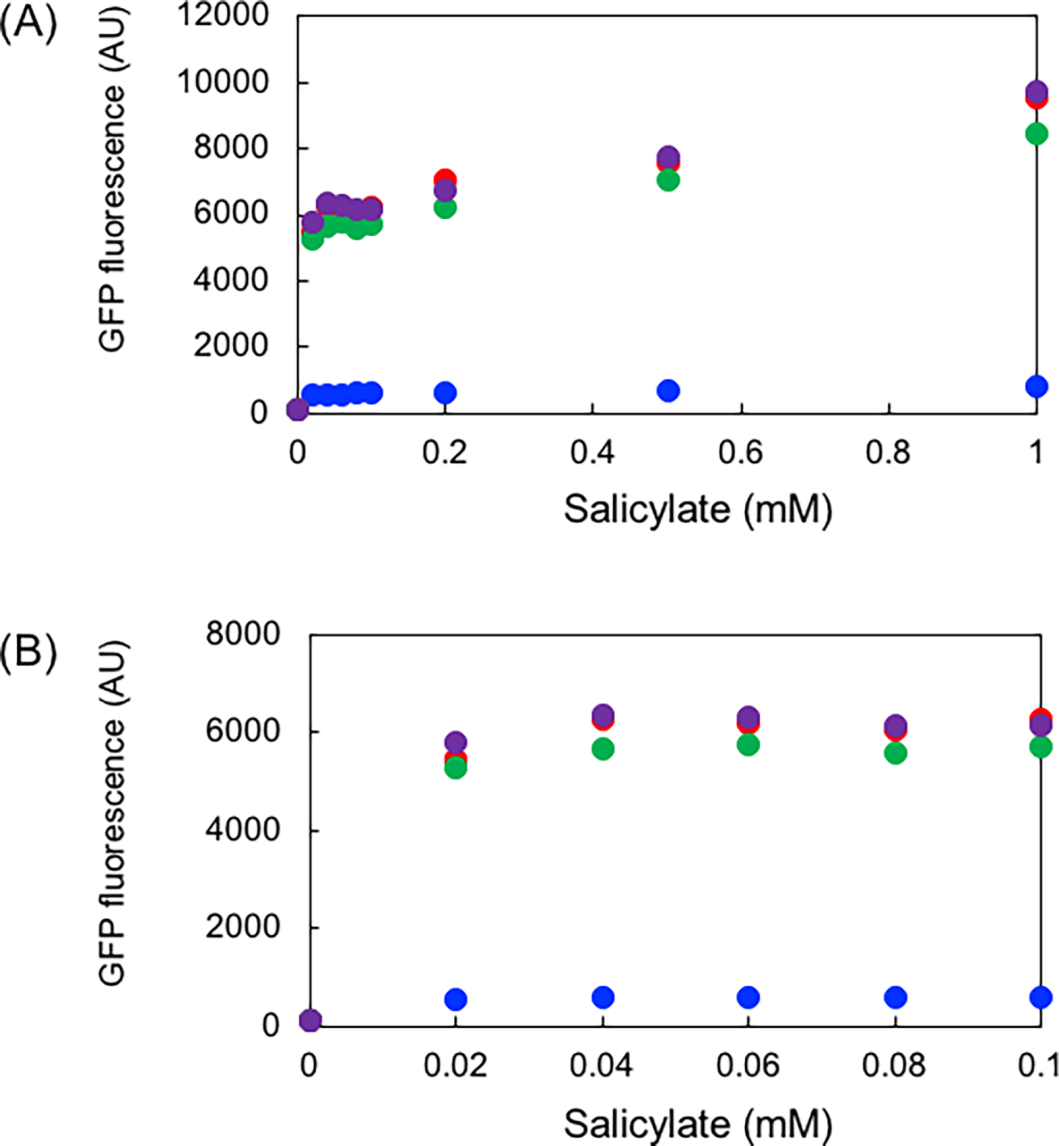
Salicylate-dependent fluorescent intensity of selected variants. (A) Salicylate concentration ranging from 0 to 1 mM. (B) Close up view of the plot in Fig 4A with salicylate concentration ranging from 0 to 0.1 mM. *E*. *coli* JM109 cells were grown in LB/Amp supplemented with the indicated concentrations of salicylate at 37°C for 16 h. *N* = 4. Symbols: blue, pSAL7A35; red, N282L(TTG); green, Q292A(GCG); and purple, V295F(TTT). Note that at 0 mM salicylate concentration, all the clones did not exhibit fluorescence and the symbols are overlapped with each other.

Among the variants obtained through the saturation mutagenesis, N282L (TTG), Q292A (GCG), and V295F (TTT), together with the wild type in the pSAL7A, pSAL7A3, and pSAL7A35 vectors were further characterized. Fig 4 illustrates the salicylate-dependence of the induction. Virtually no large differences were observed among the variants, indicating that the mutation did not alter the affinity to the compound.

#### Orthogonality test

**Fig 5:**
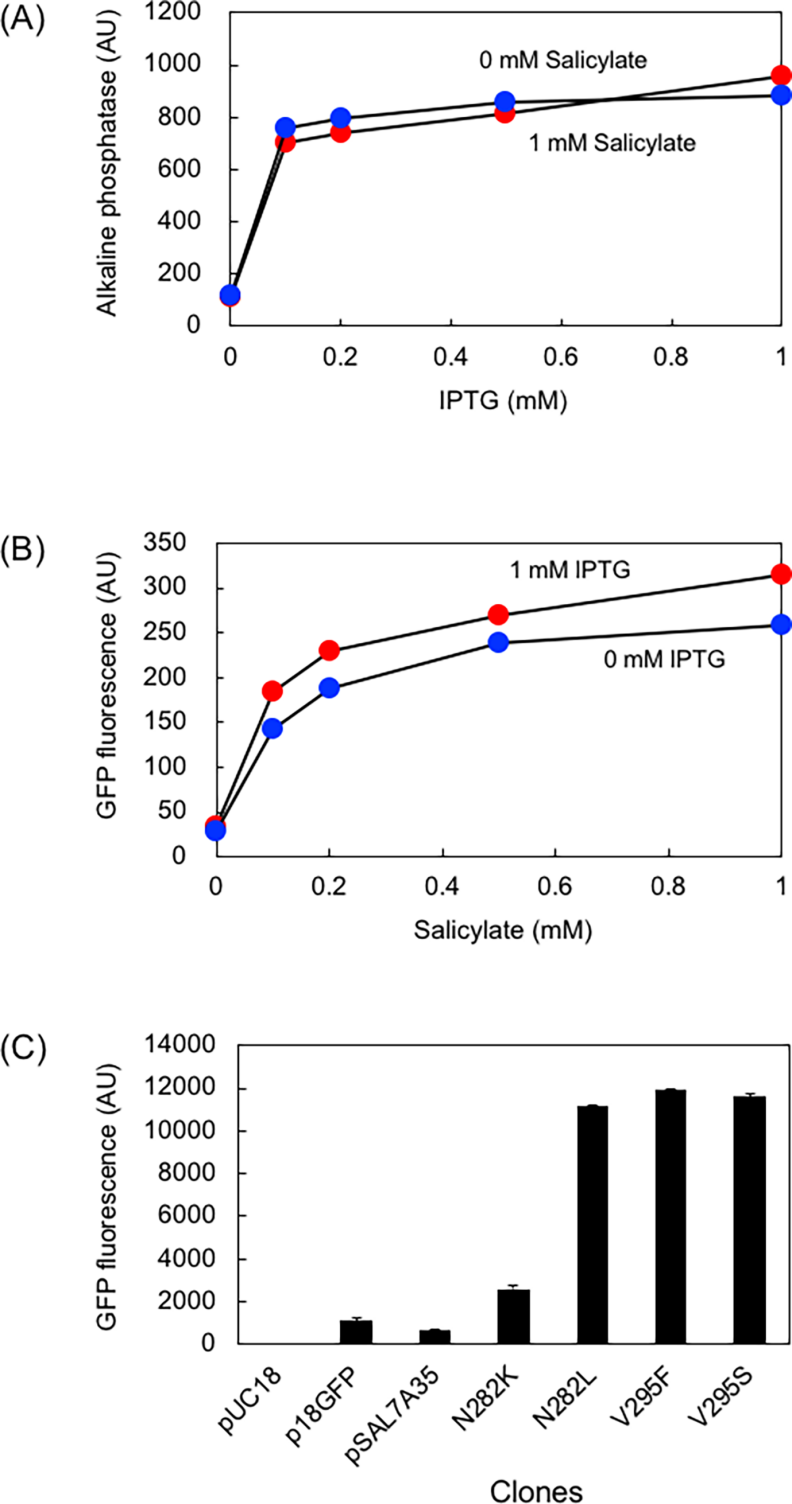
Orthogonality test. (A) IPTG-dose dependence of the alkaline phosphatase activity in the presence (blue) and absence (red) of 1 mM salicylate. (B) Salicylate-dose dependence of the GFP fluorescence in the presence (blue) and absence (red) of 1 mM IPTG. (C) Comparison of fluorescence intensity between lactose promoter-driven expression (p18GFP) and salicylate-driven induction. *E*. *coli* JM109 cells were grown in LB/Amp supplemented with either 1 mM IPTG (for p18GFP) or 1 mM salicylate (others) at 37°C for 16 h. *N* = 4.

Salicylate was not metabolized in *E*. *coli* cells and its toxicity can be negligible at 1 mM. I tested induction orthogonality with an IPTG-dependent expression system. As a reporter for the IPTG-inducible system, I used alkaline phosphatase, which was cloned in pSTV28 (p15a ori, Cm resistant). The concentration of salicylate (or IPTG) was varied from 0 to 1 mM, while the concentration of IPTG (or salicylate) was fixed at 0 or 1 mM and the GFP fluorescence and alkaline phosphatase activity were determined. As shown in Fig 5, the alkaline phosphatase activity (Fig 5A) increased with increasing IPTG concentration; however, it was mildly affected by salicylate concentration (0 or 1 mM). Similarly, the GFP fluorescence (Fig 5B) increased with increasing concentration of salicylate; however, it was mildly affected by IPTG concentration (0 or 1 mM). The rate of induction by salicylate in the improved variants was approximately 15-times that of the rate induced by lac promoter (p18GFP, GFP gene cloned in pUC18) [5] (Fig 5C).

#### Cost merit

The advantage of using salicylic acid as an induction agent is that the compound is chemically inert to *E*. *coli* metabolism (not metabolized or not produced) [9] and its transcriptional system; the compound continuously induces the expression during cultivation. Furthermore, salicylic acid has an economical advantage (~$30/kg) relative to common induction agents (arabinose, $1,500/kg; IPTG, $ 460.5/10 g) and is stable at room temperatures. Thus, the salicylic acid-inducible expression system could be an attractive option for genetic engineering experiments as well as industrial-scale production purposes.

## Supporting information

S1 Table PCR primers used in this study. (DOCX)

## References

[1] Tang SY, Fazelinia H, Cirino PC. AraC regulatory protein mutants with altered effector specificity. J Am Chem Soc. 2008; 130: 5267–71. doi: 10.1021/ja7109053 18355019 10.1021/ja7109053

[2] Kelly CL, Liu Z, Yoshihara A, Jenkinson SF, Wormald MR, Otero J, et al Synthetic chemical inducers and genetic decoupling enable orthogonal control of the rhaBAD promoter. ACS Synth Biol. 2016; 5:1136–45. doi: 10.1021/acssynbio.6b00030 27247275 10.1021/acssynbio.6b00030

[3] van der Meer JR, de Vos WM, Harayama S, Zehnder AJ. Molecular mechanisms of genetic adaptation to xenobiotic compounds. Microbiol Rev. 1992; 56:677–94. 1480115 10.1128/mr.56.4.677-694.1992 PMC372894

[4] Springael D, Top EM. Horizontal gene transfer and microbial adaptation to xenobiotics: new types of mobile genetic elements and lessons from ecological studies. Trends Microbiol. 2004; 12:53–8. 15040322 10.1016/j.tim.2003.12.010

[5] Uchiyama T, Abe T, Ikemura T, Watanabe K. Substrate-induced gene-expression screening of environmental metagenome libraries for isolation of catabolic genes. Nat Biotechnol. 2005; 23:88–93. doi: 10.1038/nbt1048 15608629 10.1038/nbt1048

[6] Uchiyama T, Miyazaki K. Product-induced gene expression, a product-responsive reporter assay used to screen metagenomic libraries for enzyme-encoding genes. Appl Environ Microbiol. 2010; 76:7029–35. doi: 10.1128/AEM.00464-10 20833789 10.1128/AEM.00464-10 PMC2976229

[7] Uchiyama T, Miyazaki K. Substrate-induced gene expression screening: a method for high-throughput screening of metagenome libraries. Methods Mol Biol. 2010; 668:153–68. doi: 10.1007/978-1-60761-823-2_10 20830562 10.1007/978-1-60761-823-2_10

[8] Uchiyama T, Miyazaki K. Metagenomic screening for aromatic compound-responsive transcriptional regulators. Plos One. 2013; 8 doi: 10.1371/journal.pone.0075795 24098725 10.1371/journal.pone.0075795 PMC3786939

[9] Ahmadi MK, Fawaz S, Jones CH, Zhang G, Pfeifer BA. Total biosynthesis and diverse applications of the nonribosomal peptide-polyketide siderophore yersiniabactin. Appl Environ Microbiol. 2015; 81:5290–8. doi: 10.1128/AEM.01373-15 26025901 10.1128/AEM.01373-15 PMC4510178

[10] Price CT, Lee IR, Gustafson JE. The effects of salicylate on bacteria. Int J Biochem Cell Biol. 2000; 32:1029–43. doi: 10.1016/S1357-2725(00)00042-X 11091136 10.1016/s1357-2725(00)00042-x

[11] Kunin CM, Hua TH, Bakaletz LO. Effect of salicylate on expression of flagella by *Escherichia coli* and *Proteus*, *Providencia*, and *Pseudomonas* spp. Infect Immun. 1995; 63:1796–9. 7729888 10.1128/iai.63.5.1796-1799.1995 PMC173226

[12] Miyazaki K, Arnold FH. Exploring nonnatural evolutionary pathways by saturation mutagenesis: Rapid improvement of protein function. J Mol Evol. 1999; 49:716–20. doi: 10.1007/PL00006593 10594172 10.1007/pl00006593

