# Supplementary material for: Molecular engineering of the salicylate-inducible transcription factor Sal7AR for orthogonal and high gene expression in Escherichia coli
Source: PLoS One. 2018 Apr 11;13(4):e0194090. doi: 10.1371/journal.pone.0194090 (PMC5894983; doi:10.1371/journal.pone.0194090)
Supplement: S1 Table — (DOCX) [file pone.0194090.s001.docx]

**S1 Table PCR Primers used in this study**

| **Primer** | **Sequence (5’ to 3’)** |
| --- | --- |
| asM13Rev  pUCPlacDel  pUCPlacDelas  orf4-10  orf4-50  orf4-90  orf4-130  orf4-170  orf4-del  TF_5UTR  TF_5UTRas  TF_Rev  EGFP_Fwd  N282X-1  N282X-2  Q292X-1  Q292X-2  V295X-1  V295X-2  Q292XV295X-1 | TTGTTATCCGCTCACAATTCC  GCCTGGGGTGCCTAATGAGTG  CACTCATTAGGCACCCCAGGC  CGGCCTGACTCGGGACCACT  CAACTCGGCATCGGCAGGTA  GCTGTAGGAAATTGGCAAAT  AGCAGATTGCTGCCGCTTTC  GGGGTTCTACGACGAGTTCC  ATTTTTTTACCGTTGTCTCCGTTGG  GAAGCCATCCTTTATTCACATTG  CAATGTGAATAAAGGATGGCTTC  TCACACTGTGGTAGGGGCATCGGCG  ATGGCTAGCAAAGGAGAAGAACTC  NNKCGTGATCCAGCCAACCTGTGGCTCAGGCAGTT  GTACTTGGCATGCCAGAACAGGT  NNKTTGCTAGTCGAGCTGTTCGCCGAT  CCTGAGCCACAGGTTGGCTGGATC  NNKGAGCTGTTCGCCGATGCCCCTACCA  TAGCAACTGCCTGAGCCACAGGTTGG  NNKTTGCTANNKGAGCTGTTCGCCGATGCCCCTAC |
